# Yoga therapy for mental resilience in technology-driven occupational settings: A qualitative systematic review and thematic synthesis

**DOI:** 10.1016/j.jaim.2026.101394

**Published:** 2026-09-16

**Authors:** Santosh Kumar Sahu, Ajit Kumar Pradhan, Priyanka Giri

**Affiliations:** a Department of Ayush, All India Institute of Medical Sciences, Bhubaneswar, Odisha, India; b Gopabandhu Ayurveda Mahavidyalaya, Puri, Odisha, India

**Keywords:** Yoga therapy, Mental resilience, Technology professionals, Occupational stress, Workplace well-being

## Abstract

Mental health concerns among individuals working in technology-driven occupational settings have emerged as a major workplace challenge. Persistent cognitive demands, prolonged screen exposure, sedentary work patterns, and psychosocial stressors are associated with burnout, sleep disturbance, emotional dysregulation, and reduced resilience. Emerging evidence suggests that yoga therapy (YT) may represent a promising mind–body approach for supporting stress regulation and psychophysiological balance in cognitively demanding work environments. This qualitative systematic review and thematic synthesis examined mechanistic and applied evidence related to yoga-based interventions in technology-related and comparable workplace populations. Literature searches were conducted across PubMed, Scopus, and Google Scholar (2010–2025). Eligible studies included randomized controlled trials, workplace intervention studies, mechanistic physiological investigations, observational studies, and relevant systematic reviews examining outcomes related to stress, autonomic function, emotional well-being, cognitive performance, and occupational health. Due to substantial heterogeneity across study designs, interventions, and outcome measures, findings were synthesized qualitatively. The included evidence suggests that YT may support autonomic regulation, heart rate variability modulation, and stress adaptation. Workplace-based interventions were generally associated with favorable trends in perceived stress reduction, sleep quality, emotional well-being, cognitive functioning, and occupational satisfaction. Brief yoga-based micro-practices also appeared feasible for workplace stress management and attentional recovery. Classical yogic concepts, including sattva and prāṇa vāta regulation, provide an interpretive framework for understanding the multidimensional effects of YT. Overall, yoga-based interventions may represent a promising integrative strategy for resilience-related outcomes in technology-driven occupational settings. However, findings should be interpreted cautiously because of methodological heterogeneity and limited occupation-specific evidence. This review was prospectively registered with PROSPERO (CRD420251240676).

## 1 Introduction

The rapid expansion of the technology sector has substantially transformed modern occupational environments, generating new patterns of psychological strain and work-related stress. Professionals working in technology-driven settings are frequently exposed to sustained cognitive demands, prolonged screen engagement, multitasking, digital surveillance, and persistent time pressure, which are associated with anxiety, sleep disturbance, emotional exhaustion, and burnout [1]. In the post-pandemic era, the convergence of remote work, digital overload, reduced work–life boundaries, and job insecurity has further intensified mental fatigue and psychosocial stress among employees. Consequently, mental resilience the capacity to adapt positively to stress and recover from adversity has emerged as a critical determinant of occupational well-being and long-term work sustainability [2].

Yoga therapy (YT) may be broadly defined as the individualized and evidence-informed application of yogic practices to address physical, psychological, and psychosocial health challenges. According to the International Association of Yoga Therapists (IAYT), YT involves the professional application of principles and practices derived from the yoga tradition to support health, adaptive functioning, and therapeutic outcomes within individualized or group-based contexts. Unlike recreational or fitness-oriented yoga programs that primarily emphasize physical flexibility and exercise, YT adopts a therapeutic and goal-oriented framework integrating āsana (postural practices), prāṇāyāma (breath regulation), dhyāna (meditation), relaxation techniques, and behavioral self-regulation strategies tailored to specific health conditions or occupational stress profiles. In occupational settings, yoga-based interventions are increasingly being adapted to support stress management, emotional regulation, sleep quality, cognitive recovery, and autonomic balance within demanding professional environments [3,4].

In the present review, the term “technology professionals” refers broadly to individuals engaged in predominantly digital, computer-mediated, and cognitively intensive occupational roles, including software developers, information technology (IT) employees, programmers, cybersecurity personnel, data analysts, remote knowledge workers, and related technology-sector professionals. These work environments are typically characterized by prolonged sedentary behavior, sustained attentional demands, rapid task switching, continuous digital connectivity, and deadline-driven workflows. However, because occupation-specific yoga research focusing exclusively on technology-sector employees remains limited, the present review also incorporates evidence from comparable cognitively demanding workplace populations including office employees, healthcare professionals, educators, and other stress-exposed occupational groups where similar psychophysiological stress patterns may occur. Throughout the review, distinctions are made between direct evidence derived from technology-related occupational settings and extrapolative evidence obtained from broader workplace populations.

Emerging evidence suggests that yoga-based interventions may support psychophysiological regulation and resilience-related outcomes in high-stress occupational populations [5,6]. Beyond physical exercise, YT incorporates multidimensional behavioral and contemplative practices intended to influence emotional regulation, self-awareness, attentional stability, and adaptive stress responses. Several studies have reported associations between yoga practice and reductions in perceived stress, sleep disturbance, anxiety symptoms, and occupational fatigue, as well as improvements in emotional well-being and executive functioning [7,8]. These observations are particularly relevant in technology-driven workplaces, where chronic cognitive load and limited recovery opportunities may contribute to autonomic dysregulation, impaired concentration, and reduced occupational well-being.

The technology sector represents one of the most cognitively demanding occupational environments, requiring prolonged attention, continuous information processing, multitasking, and repetitive interactions with digital interfaces. Such conditions are increasingly associated with musculoskeletal discomfort, sleep disturbances, emotional exhaustion, and impaired mental health in the working population [9,10]. Contemporary occupational stress models provide useful frameworks for understanding these dynamics. According to the Job Demand–Control model, stress-related outcomes are more likely when high occupational demands coexist with limited autonomy and insufficient recovery opportunities [11]. Similarly, the Effort–Reward Imbalance model proposes that chronic stress emerges when sustained occupational effort is not matched by adequate recognition, support, or compensation [12]. Among technology professionals, these psychosocial stressors frequently coexist and may collectively contribute to burnout, reduced resilience, and compromised psychological functioning.

From an occupational health perspective, yoga-based interventions may offer feasible and scalable approaches for supporting stress adaptation and recovery in demanding work environments. Workplace studies have reported favorable trends in perceived stress reduction, emotional well-being, musculoskeletal comfort, and sleep quality following structured yoga-based programs [13,14]. Randomized controlled trials involving university employees and healthcare personnel have also demonstrated improvements in stress-related and occupational outcomes following 6–8-week interventions [15]. In addition, systematic reviews and meta-analyses suggest that workplace yoga interventions may contribute to improved mood, stress management, and well-being across diverse occupational populations [16,17].

Technology-driven occupations are characterized by prolonged sedentary work patterns and limited opportunities for physiological recovery during work hours. Evidence from workplace micro-break research suggests that brief rest intervals of ≤10 min may support attentional restoration, vigor, and work performance [18]. In this context, short yoga-based “micro-practices” integrated into workplace routines may represent practical approaches for stress management and cognitive recovery without substantially disrupting productivity. Mechanistic investigations further suggest that yoga-based practices may influence stress-related psychophysiology through pathways associated with autonomic regulation, heart rate variability modulation, neuroendocrine adaptation, and interoceptive awareness [[19], [20], [21], [22]]. Collectively, these findings provide a biologically plausible and occupationally relevant framework for understanding the potential role of YT in resilience-related outcomes.

Therefore, this review aims to qualitatively synthesize mechanistic and applied evidence related to YT and resilience-related outcomes in technology-driven occupational settings. This review further seeks to integrate occupational, psychophysiological, and yogic conceptual perspectives while outlining practical implications for workplace mental health promotion and integrative occupational well-being strategies.

## 2 Methods

### 2.1 Search strategy

A comprehensive literature search was conducted across PubMed, Scopus, and Google Scholar for studies published between January 2010 and August 2025. The present review was conducted and reported in accordance with the Preferred Reporting Items for Systematic Reviews and Meta-Analyses (PRISMA-2020) guidelines to improve methodological transparency and reproducibility [23]. The search strategy combined controlled vocabulary terms and free-text keywords related to YT, occupational stress, resilience, workplace well-being, autonomic regulation, and technology-driven occupational settings. Boolean operators (“AND,” “OR”) were applied in various combinations to optimize search sensitivity and specificity across databases. Search syntax was adapted according to database-specific indexing requirements.

Representative search terms included: (“yoga therapy” OR yoga OR prāṇāyāma OR meditation) AND (“occupational stress” OR burnout OR resilience OR workplace stress) AND (“technology professionals” OR IT employees OR desk workers OR cognitively demanding occupations). The complete reproducible search strategies, search syntax, and applied filters for all databases are provided in Supplementary Table S1. In addition, reference lists of eligible studies and relevant reviews were manually screened to identify potentially relevant publications not retrieved during the electronic database search.

### 2.2 Review design and rationale

The present study was designed as a qualitative systematic review with thematic synthesis of mechanistic and applied evidence related to YT and resilience-related outcomes in occupational settings. A mixed-evidence review design was intentionally adopted because the available literature in this emerging interdisciplinary field remains methodologically heterogeneous and includes randomized controlled trials, workplace intervention studies, mechanistic physiological investigations, observational studies, and systematic reviews/meta-analyses. Restricting the review exclusively to randomized quantitative studies would have substantially limited the ability to synthesize broader neurophysiological, cognitive-behavioral, autonomic, and occupational evidence relevant to technology-driven work environments.

Accordingly, this review aimed to integrate complementary forms of evidence to provide a broader conceptual, mechanistic, and translational understanding of yoga-based interventions within cognitively demanding occupational settings. To improve methodological transparency, included studies were categorized according to evidence type and synthesized thematically across mechanistic, psychophysiological, cognitive-behavioral, and workplace-related domains. Because of substantial heterogeneity in study populations, intervention protocols, outcome measures, and methodological designs, formal quantitative meta-analysis was not considered methodologically appropriate.

### 2.3 Eligibility criteria

Studies were included if they met the following criteria:

a) Population: Adults engaged in occupational or cognitively demanding work environments, including technology professionals, IT employees, office workers, healthcare professionals, educators, and comparable occupational populations exposed to chronic workplace stress.
b) Intervention: Yoga-based or yoga-integrated interventions including āsana, prāṇāyāma, meditation, mindfulness-based yoga practices, relaxation techniques, or structured workplace yoga programs.
c) Outcomes: Studies reporting at least one psychological, physiological, autonomic, neuroendocrine, cognitive, or occupational outcome related to stress regulation, emotional well-being, resilience-related outcomes, burnout, sleep quality, cognitive performance, or autonomic balance.
d) Study design: Randomized controlled trials, quasi-experimental studies, observational studies, physiological/mechanistic investigations, and systematic reviews/meta-analyses.
e) Publication characteristics: Peer-reviewed English-language publications published between January 2010 and August 2025.

### 2.4 Study selection

**Fig. 1:**
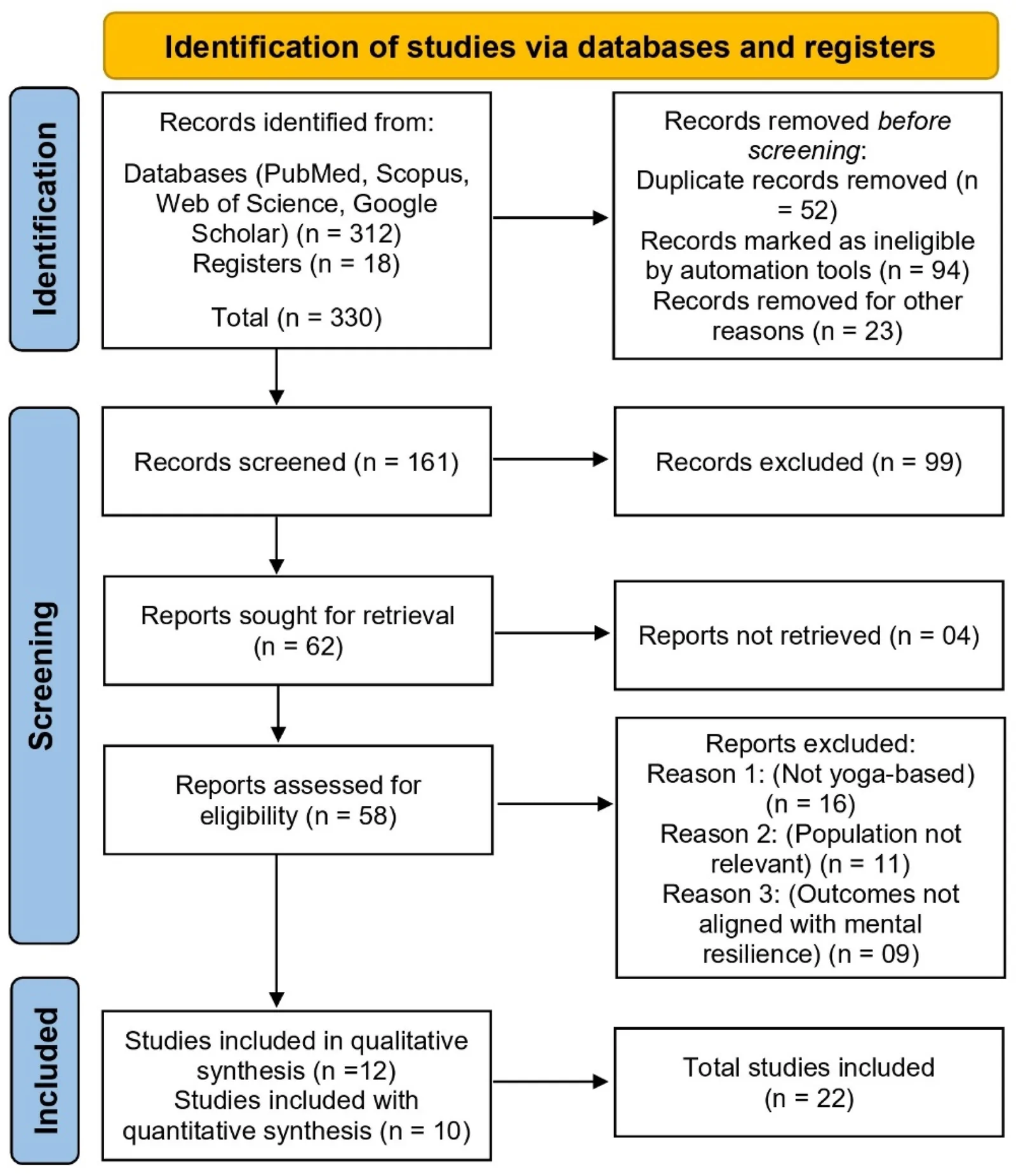
PRISMA flow chart of the literature search and study selection process.

All identified records were exported and screened in a stepwise manner according to PRISMA 2020 recommendations. After removal of duplicate records, titles and abstracts were independently screened for relevance using predefined eligibility criteria. Full-text articles deemed potentially eligible were subsequently assessed in detail. Studies were excluded during full-text assessment if they: a) did not involve yoga-based interventions, b) lacked occupational, resilience-related, or stress-related outcomes, c) focused exclusively on physical fitness outcomes without psychophysiological relevance, d) represented non-empirical publications or commentaries, or e) did not satisfy methodological or population eligibility criteria. Disagreements regarding study eligibility were resolved through discussion and consensus between reviewers. The complete screening and study-selection process is summarized in the PRISMA 2020 flow diagram (Fig. 1).

The database search identified 330 records. Following duplicate removal and title–abstract screening, 58 full-text articles were assessed for eligibility. Of these, 22 studies met all predefined inclusion criteria and were included in the final qualitative thematic synthesis. Common reasons for exclusion during full-text review included absence of yoga-based intervention, lack of occupational or resilience-related outcomes, insufficient psychophysiological relevance, and non-empirical study design.

### 2.5 Data extraction and synthesis

Data extraction was independently performed by two reviewers to improve methodological consistency and minimize interpretive bias. Extracted variables included author details, publication year, study design, population characteristics, occupational relevance, intervention type and duration, outcome measures, and principal findings. Discrepancies were resolved through discussion and consensus. To improve synthesis transparency, included studies were additionally categorized according to evidence type: 1) randomized controlled workplace interventions, 2) mechanistic physiological investigations, 3) observational or pilot occupational studies, and 4) systematic reviews/meta-analyses.

The findings were synthesized thematically across two primary domains:

a) Mechanistic evidence: Studies examining neuroendocrine regulation, autonomic modulation, heart rate variability, vagal activity, interoceptive awareness, and neurocognitive control mechanisms.
b) Applied workplace outcomes: Studies evaluating perceived stress, burnout, sleep quality, emotional well-being, cognitive functioning, workplace satisfaction, and related occupational outcomes.

Where appropriate, relevant classical yogic concepts including manas, sattva, and prāṇa vāta were incorporated to facilitate conceptual alignment between traditional yogic frameworks and contemporary psychophysiological interpretation.

Because included studies differed substantially in design, population characteristics, intervention formats, and measured outcomes, findings were synthesized qualitatively using a thematic integrative approach rather than statistical pooling. Mechanistic physiological studies informed interpretation of autonomic and neuroendocrine regulation, workplace intervention studies contributed applied occupational evidence, observational investigations provided contextual psychophysiological insights, and systematic reviews/meta-analyses contextualized broader trends across stress-related and occupational populations. Themes were iteratively developed through comparative analysis of convergent findings across these evidence categories.

### 2.6 Quality appraisal

Methodological quality and potential sources of bias were assessed narratively according to study design characteristics, including sample-size adequacy, intervention clarity, control comparability, outcome reporting, duration of follow-up, and occupational relevance. Randomized studies were evaluated with attention to allocation procedures, control conditions, and reporting transparency, whereas observational and mechanistic investigations were assessed for methodological rigor, external validity, and interpretive limitations. Given the heterogeneity of evidence types included in this review, a formal pooled risk-of-bias scoring framework was not considered uniformly applicable. Instead, methodological limitations and quality considerations were integrated qualitatively into the thematic interpretation of findings.

## 3 Results and thematic synthesis

A total of twenty-two studies representing diverse evidence categories including randomized controlled trials, workplace intervention studies, mechanistic physiological investigations, observational studies, and systematic reviews/meta-analyses were included in the final qualitative thematic synthesis (Table 1). To improve occupational transparency, studies were additionally classified according to their relevance to technology-sector populations, comparable workplace settings, or broader stress-exposed populations. The included literature was also categorized according to evidence type, comprising: (1) workplace intervention studies, (2) mechanistic physiological investigations, (3) clinical and observational studies, and (4) secondary evidence sources including systematic reviews and meta-analyses.

**Table 1:** Characteristics, occupational relevance, and evidence categories of included studies examining yoga-based interventions for mental resilience and stress regulation.

Sl. No.	Author(s), Year	Evidence Category	Study Design	Population/Sample Size	Occupational Relevance	Intervention Type	Duration/Frequency	Main Outcomes/Key Findings
1	Hartfiel et al., 2011 [1]	Workplace intervention	Randomized controlled trial	University employees (n = 48)	Comparable workplace population	Weekly Dru Yoga sessions	60 min/week for 6 weeks	Improved resilience-related and workplace well-being outcomes.
2	Hartfiel et al., 2012 [2]	Workplace intervention	Pragmatic controlled trial	Local government employees (n = 37)	Comparable workplace population	Group Dru Yoga + home practice	8 weeks	Reduced perceived stress and back pain.
3	Della Valle et al., 2020 [6]	Secondary evidence	Systematic review and meta-analysis	Multiple occupational sectors (12 studies)	Secondary evidence source	Workplace yoga interventions	4–12 weeks (varied)	Reported reductions in occupational stress outcomes.
4	Albulescu et al., 2022 [8]	Secondary evidence	Systematic review and meta-analysis	Occupational populations (22 studies)	Secondary evidence source	Micro-break interventions	≤10 min	Improved vigor and reduced fatigue.
5	Telles et al., 2011 [7]	Mechanistic study	Experimental physiological study	Healthy adults (n = 38)	General stress-exposed/mechanistic population	Yogic breathing practices	Single session	Improved HRV indices and autonomic markers.
6	Yoshihara et al., 2014 [10]	Clinical observational study	Prospective single-arm study	Women with stress-related symptoms (n = 24)	General stress-exposed population	Integrated yoga practice	12 weeks	Reduced psychological distress and somatization.
7	Pascoe et al., 2017 [11]	Secondary evidence	Meta-analysis	Adults under stress (42 studies)	Secondary evidence source	Yoga and mindfulness interventions	Varied	Reduced physiological stress markers.
8	Gerritsen & Band, 2018 [12]	Mechanistic review	Narrative physiological review	Not applicable	General mechanistic evidence	Slow breathing and contemplative practices	Not applicable	Discussed vagal and baroreflex mechanisms.
9	Tang et al., 2015 [13]	Mechanistic review	Neuroscientific review	Not applicable	General mechanistic evidence	Mindfulness meditation	Varied	Described neural regulation mechanisms.
10	Deepeshwar & Budhi, 2022 [14]	Mechanistic study	Experimental study	Healthy male volunteers (n = 40)	General stress-exposed/mechanistic population	Slow yogic breathing	Short-term	Improved working memory and cardiac activity.
11	Mehling et al., 2012 [15]	Psychophysiological study	Instrument validation study	Adults from stress-related studies (n = 325)	General stress-exposed population	Interoceptive awareness assessment	Cross-sectional	Interoception associated with emotional regulation.
12	Parajuli et al., 2021 [17]	Workplace intervention	Pilot intervention study	Female nursing professionals (n = 33)	Comparable workplace population	Integrated yoga sessions	4 weeks	Improved stress and sleep quality.
13	Metri et al., 2023 [18]	Workplace intervention	Randomized controlled study	Female teachers (n = 50)	Comparable workplace population	Integrated Yoga Therapy program	6 weeks	Reduced stress, anxiety, fatigue, and pain outcomes.
14	Nivethitha et al., 2017 [22]	Mechanistic study	Physiological experimental study	Healthy adults (n = 16)	General stress-exposed/mechanistic population	Bhrāmarī prāṇāyāma	Single session	Increased parasympathetic activity.
15	Pal et al., 2004 [20]	Mechanistic study	Experimental physiological study	Healthy volunteers (n = 60)	General stress-exposed/mechanistic population	Yogic breathing exercises	Short-term	Improved autonomic balance.
16	Thayer et al., 2012 [24]	Secondary evidence	Meta-analysis	Neuroimaging and HRV studies (8 studies)	Secondary evidence source	Contemplative practices	Varied	Identified HRV–emotion regulation associations.
17	Rocha et al., 2012 [25]	Clinical intervention study	Randomized controlled trial	Women with stress symptoms (n = 36)	General stress-exposed population	Yoga-based stress reduction	6 months	Improved physiological and psychological parameters.
18	Korkmaz et al., 2024 [21]	Workplace intervention	Randomized clinical trial	Physicians (n = 129)	Comparable workplace population	Sudarshan Kriya Yoga	8 weeks	Reduced burnout and cortisol levels.
19	Cheema et al., 2011 [26]	Workplace intervention	Controlled workplace trial	Office employees/sedentary workers (n = 56)	Direct technology-sector evidence	Office-based yoga intervention	10 weeks	Improved HRV and reduced workplace stress.
20	Telles et al., 2017 [27]	Mechanistic study	Experimental crossover study	Experienced yoga practitioners (n = 15)	General mechanistic evidence	Alternate nostril breathing	Single session	Improved vigilance and attentional performance.
21	Schleinzer et al., 2024 [16]	Secondary evidence	Systematic review and meta-analysis	Adults with elevated stress (13 studies)	Secondary evidence source	Yoga-based stress reduction	Varied	Supported yoga as a stress-management approach.
22	Gothe et al., 2016 [19]	Mechanistic study	Pilot neurocognitive study	Sedentary adults (n = 118)	General stress-exposed population	Yoga and mindfulness training	8 weeks	Improved executive function and stress regulation.

Across studies, intervention formats ranged from brief yoga-based micro-practices integrated within workplace routines to structured group-based programs lasting between 4 and 12 weeks. Overall, the evidence demonstrated generally favorable trends in perceived stress reduction, autonomic regulation, heart rate variability (HRV), sleep quality, emotional well-being, and resilience-related outcomes. However, methodological heterogeneity and variability in study quality warrant cautious interpretation of findings. A summary of methodological quality considerations, limitations, and potential sources of bias across included studies is presented in Table 2.

**Table 2:** Methodological quality, limitations, and potential sources of bias across included studies.

Sl. No.	Author(s), Year	Study Design	Main Methodological Limitations	Quality/Risk of Bias Considerations
1	Hartfiel et al., 2011 [1]	Randomized controlled trial	Small sample size; short intervention duration; limited occupational diversity	Moderate risk of bias due to small sample and short follow-up
2	Hartfiel et al., 2012 [2]	Pragmatic workplace trial	Reliance on self-reported outcomes; limited long-term follow-up	Moderate risk of reporting and expectancy bias
3	Della Valle et al., 2020 [6]	Systematic review and meta-analysis	Considerable heterogeneity across included workplace interventions	Moderate heterogeneity-related bias
4	Albulescu et al., 2022 [8]	Systematic review and meta-analysis	Included diverse micro-break interventions beyond yoga-specific protocols	Moderate indirectness of evidence
5	Telles et al., 2011 [7]	Experimental physiological study	Single-session intervention; healthy non-clinical population	Limited external validity and long-term inference
6	Yoshihara et al., 2014 [10]	Prospective single-arm study	Lack of control group; small sample size	High risk of selection and performance bias
7	Pascoe et al., 2017 [11]	Meta-analysis	Variability in intervention protocols and stress biomarkers	Moderate heterogeneity and publication bias risk
8	Gerritsen & Band, 2018 [12]	Narrative review	Non-systematic review methodology; theoretical interpretation	High interpretive bias risk
9	Tang et al., 2015 [13]	Neuroscientific review	Primarily mindfulness-focused evidence; indirect occupational applicability	Moderate indirectness and conceptual heterogeneity
10	Deepeshwar & Budhi, 2022 [14]	Experimental study	Small homogeneous sample; short-term intervention only	Moderate generalizability limitations
11	Mehling et al., 2012 [15]	Instrument validation study	Cross-sectional design; not an intervention study	Limited causal inference
12	Parajuli et al., 2021 [17]	Pilot workplace study	Small pilot sample; absence of long-term follow-up	Moderate-to-high risk of bias
13	Metri et al., 2023 [18]	Randomized controlled trial	Female-only sample; occupation-specific population	Moderate external validity limitations
14	Nivethitha et al., 2017 [22]	Physiological experimental study	Acute intervention only; healthy participants	Limited applicability to occupational stress populations
15	Pal et al., 2004 [20]	Experimental physiological study	Older study design; limited occupational relevance	Moderate methodological limitations
16	Thayer et al., 2012 [24]	Meta-analysis	Neuroimaging methodological heterogeneity	Moderate heterogeneity risk
17	Rocha et al., 2012 [25]	Randomized controlled trial	Small sample size; female-only participants	Moderate generalizability limitations
18	Korkmaz et al., 2024 [21]	Randomized clinical trial	Specific occupational group (physicians); intervention complexity	Moderate intervention-related heterogeneity
19	Cheema et al., 2011 [26]	Controlled workplace trial	Limited sample size; incomplete long-term assessment	Moderate risk of attrition and reporting bias
20	Telles et al., 2017 [27]	Experimental crossover study	Single-session design; experienced yoga practitioners only	Limited generalizability
21	Schleinzer et al., 2024 [16]	Systematic review and meta-analysis	Considerable variation in intervention types and outcome measures	Moderate heterogeneity and publication bias risk
22	Gothe et al., 2016 [19]	Pilot neurocognitive study	Pilot-scale design; limited occupational specificity	Moderate preliminary-evidence limitations

Among the included studies, only a limited number directly investigated technology-sector or office-based employee populations. Several mechanistic and workplace-related findings were extrapolated from broader occupational or stress-exposed groups, including healthcare professionals, educators, sedentary adults, and general workplace populations. Consequently, occupation-specific interpretations relevant to technology-driven work environments should be considered cautiously. In addition, several studies were limited by relatively small sample sizes, short intervention durations, reliance on self-reported outcomes, and limited long-term follow-up, factors that may affect interpretive strength and generalizability.

### 3.1 Neuroendocrine evidence

Neuroendocrine regulation plays a central role in maintaining physiological homeostasis during stress exposure. Chronic occupational strain characterized by excessive cognitive load, prolonged working hours, and emotional exhaustion has been associated with dysregulation of the hypothalamic–pituitary–adrenal (HPA) axis, including elevated cortisol secretion, impaired feedback inhibition, and increased inflammatory activity [11,16]. Sustained HPA-axis activation, commonly observed in occupational burnout, may contribute to fatigue, mood instability, sleep disturbance, and reduced adaptive capacity [28].

Several investigations suggest that yoga-based interventions may support neuroendocrine regulation and stress adaptation. One study reported that yoga and mindfulness-based interventions were associated with reductions in cortisol levels and improvements in immune-related parameters across occupational populations [11]. Similarly, randomized workplace studies involving healthcare personnel and university employees demonstrated improvements in sleep quality, perceived stress, and affective regulation following 8–12-week yoga-based interventions [15,25]. A pilot study involving nursing professionals also reported reductions in perceived stress and improved sleep quality following integrated YT programs [17].

From a broader occupational health perspective, workplace yoga interventions incorporating āsana, prāṇāyāma, and relaxation practices have been associated with improvements in emotional well-being, perceived stress, sleep quality, and quality of life among employed adults [18]. Neuroimaging evidence further suggests increased activation within prefrontal and insular cortical regions involved in emotional regulation, self-awareness, and interoceptive processing among experienced yoga practitioners [13,18]. These observations may help explain the resilience-related and affective benefits reported across workplace populations.

Within classical yogic frameworks, such adaptive psychophysiological regulation may conceptually correspond to the balance of prāṇa vāta and the cultivation of sattva, one of the three guṇas associated with psychological harmony, lucidity, and emotional stability. Although these traditional concepts should not be interpreted as direct biomedical equivalents, they provide a broader interpretive framework for understanding multidimensional stress regulation and adaptive functioning.

### 3.2 Autonomic regulation

Autonomic imbalance characterized by sympathetic overactivation and reduced parasympathetic activity represents an important physiological correlate of chronic occupational stress. HRV, a widely used biomarker of autonomic flexibility, has been associated with emotional regulation, cardiovascular adaptability, and resilience-related functioning [24]. Technology-driven occupational environments are frequently characterized by prolonged sedentary work, sustained attentional demands, and chronic cognitive stress, conditions associated with reduced HRV and persistent sympathetic dominance [14,29].

Several studies reported associations between yoga practice and improvements in autonomic regulation. Slow breathing techniques, including bhrāmarī, nāḍī śodhana, and alternate-nostril breathing, are associated with increased parasympathetic activity, improved baroreflex sensitivity, and reductions in heart rate and blood pressure [20,22]. A controlled workplace study involving sedentary employees demonstrated improvements in HRV following a structured office-based yoga program [26]. Another investigation reported enhanced vigilance and reduced systolic blood pressure during alternate-nostril breathing practices [27].

Collectively, these findings suggest that structured yoga-based interventions, including brief workplace “micro-practices,” may support autonomic adaptability and stress recovery within cognitively demanding occupational settings. From a traditional yogic perspective, balanced prāṇa flow is considered essential for emotional and mental stability. The observed enhancement of vagal activity through mindful breathing practices may therefore represent a convergent psychophysiological mechanism underlying resilience-related outcomes.

### 3.3 Cognitive behavioral mechanisms

Cognitive and behavioral functioning represents another important domain through which yoga-based interventions may influence resilience-related outcomes. Occupational performance within technology-driven environments depends heavily on executive functions such as sustained attention, working memory, emotional inhibition, and cognitive flexibility, capacities that are particularly vulnerable to chronic stress exposure.

Functional neuroimaging and electrophysiological investigations suggest that yoga and mindfulness-based practices may enhance prefrontal activation, reduce amygdala hyperreactivity, and increase alpha and theta electroencephalographic coherence, thereby supporting attentional regulation and emotional processing [13,18]. A pilot study reported improvements in executive functioning and working memory following yoga practice, potentially mediated through reductions in physiological stress responses [19]. Another mechanistic investigation found that slow yogic breathing improved working memory performance and cardiac efficiency during cognitively demanding tasks [14]. Workplace-based randomized studies additionally reported improvements in emotional well-being, perceived stress, and quality of life following integrated yoga-based interventions [18].

Yoga practice has also been associated with enhanced interoceptive awareness, defined as the conscious perception of internal bodily signals mediated through insular and anterior cingulate cortical networks. Increased interoceptive sensitivity, as assessed using the Multidimensional Assessment of Interoceptive Awareness (MAIA), has been correlated with improved emotional regulation and lower stress reactivity [15]. For professionals engaged in prolonged screen-based work, strengthening interoceptive awareness may help reduce emotional exhaustion and cognitive overload.

Behaviorally, yoga-based interventions may support adaptive coping processes grounded in non-reactivity, attentional stability, and mindful self-regulation. These mechanisms conceptually parallel the yogic constructs of samatva (equanimity) and vairāgya (non-attachment), which emphasize emotional balance and cognitive steadiness under challenging circumstances. Collectively, these findings suggest that yoga-based practices may support psychological flexibility and adaptive functioning relevant to occupational resilience.

### 3.4 Workplace application and feasibility

Beyond individual physiological outcomes, yoga-based interventions have demonstrated potential organizational and psychosocial relevance within occupational settings. Workplace programs incorporating brief structured yoga sessions ranging from 15 to 30 min once or twice weekly were associated with reductions in perceived stress, musculoskeletal discomfort, and back pain, alongside improvements in posture and general well-being [6,8,30]. Systematic reviews and meta-analyses further suggest that workplace yoga interventions may support stress management and mood-related outcomes without substantially disrupting productivity [7,16].

Recent workplace investigations also indicate that participation in structured multi-week yoga programs may contribute to reductions in anxiety symptoms, improvements in sleep quality, and enhanced occupational well-being among working adults [18]. Similarly, micro-break interventions lasting less than 10 min and combined with mindful breathing or stretching practices were associated with improved vigor, reduced fatigue, and enhanced task performance [8].

From an occupational-health perspective, integrating yoga-based interventions into workplace wellness frameworks may offer dual benefits by supporting employee well-being while potentially improving workplace functioning and recovery [31]. Such approaches may additionally contribute to normalization of stress-management practices and broader discussions surrounding mental health in professional environments. Within yogic psychology, these collective outcomes conceptually align with cultivation of a sāttvika mental state characterized by clarity, emotional balance, and harmonious interpersonal functioning.

### 3.5 Cardiovascular and baroreflex function

Cardiovascular regulation represents another important physiological mechanism through which yoga-based practices may support resilience-related outcomes. Controlled breathing practices, particularly slow breathing at approximately 0.1 Hz (around six breaths per minute), have been associated with improvements in baroreflex sensitivity, respiratory sinus arrhythmia, and blood pressure regulation [17,22]. These effects may reflect improved autonomic–cardiovascular coupling and enhanced stress-recovery capacity.

For professionals engaged in prolonged sedentary work and sustained digital engagement, regular slow prāṇāyāma practice may provide a feasible non-pharmacological strategy for supporting cardiovascular adaptability and reducing allostatic load [14,20,26]. One randomized controlled trial reported improvements in both physiological and psychological parameters following six months of regular yoga practice, suggesting potential integrative benefits for cardiometabolic and emotional health [25].

Taken together, the enhancement of cardiovascular and baroreflex function provides a biologically plausible framework linking breath-regulation practices with psychophysiological resilience. These observations support the broader hypothesis that yoga-based interventions may influence adaptive functioning through interconnected autonomic, cardiovascular, and emotional-regulatory pathways.

## 4 Discussion

Given the methodological heterogeneity and predominance of short-term intervention studies, the present findings should be interpreted cautiously and viewed as suggestive rather than definitive causal evidence. Because occupational yoga research remains interdisciplinary and methodologically diverse, the present review adopted an integrative thematic synthesis approach combining mechanistic, psychophysiological, and workplace-oriented evidence relevant to resilience and stress regulation. Accordingly, the present synthesis should be interpreted as a qualitative overview of emerging occupational and mechanistic evidence rather than a definitive quantitative efficacy evaluation.

Overall, the findings suggest that YT may offer a multidimensional approach for supporting resilience-related outcomes in technology-driven occupational settings. Across mechanistic and applied studies, yoga-based interventions were associated with favorable trends in autonomic regulation, stress adaptation, emotional regulation, executive functioning, and workplace well-being. Collectively, these observations indicate that YT may help address both physiological dysregulation and cognitive–emotional overload commonly associated with cognitively demanding professional environments.

### 4.1 Integration with prior literature

The present findings are broadly consistent with prior literature examining yoga-based interventions in occupational and stress-related contexts. Mechanistic evidence suggests that YT may influence autonomic nervous system functioning through increased vagal activity and improvements in high-frequency heart rate variability, thereby supporting autonomic flexibility and stress adaptation [11,32]. Several studies also reported associations between yoga practice and reduced hypothalamic–pituitary–adrenal (HPA) axis hyperactivation, reflected in favorable cortisol-related outcomes and improved psychophysiological regulation [11,16].

These physiological observations align with neurocognitive investigations reporting improvements in attentional control, emotional regulation, and working-memory performance following prāṇāyāma, meditation, and integrated yoga-based practices [13,14]. Applied workplace studies similarly reported reductions in perceived stress, burnout, musculoskeletal discomfort, and techno-strain, alongside improvements in emotional well-being, job satisfaction, and occupational functioning among employees participating in yoga-based programs [1,6,8,26].

When interpreted within occupational stress frameworks relevant to digital work environments, these findings are particularly applicable to technology-driven professions characterized by information overload, rapid task switching, prolonged sedentary work, and continuous digital connectivity [33]. Collectively, the evidence supports the broader hypothesis that yoga-based interventions may contribute to resilience-related outcomes through interconnected autonomic, cognitive, behavioral, and occupational pathways.

### 4.2 Interpretive framework and theoretical implications

From an integrative perspective, the potential benefits of YT may derive from its simultaneous engagement of body, breath, attention, and behavior. Regular practice may contribute to improvements in emotional regulation, executive functioning, attentional stability, and stress adaptation through interconnected autonomic and neuroendocrine mechanisms. This multidimensional mode of action distinguishes yoga-based interventions from conventional stress-management approaches that often target isolated physiological or psychological pathways [34].

The cultivation of self-awareness, embodied attention, and cognitive flexibility through yoga practice also aligns with contemporary psychobiological models of resilience emphasizing neuroplasticity, interoceptive awareness, and adaptive self-regulation [35]. In occupational contexts, workplace-based yoga interventions may additionally foster social cohesion, emotional balance, and recovery-oriented workplace cultures that support organizational well-being and productivity.

Classical yogic constructs including sattva, prāṇa vāta regulation, and samatva (equanimity) may offer useful conceptual correspondences for interpreting resilience-related processes within a broader biopsychospiritual framework [36]. In the present review, sattva is interpreted specifically as one of the three guṇas associated with psychological balance, harmony, lucidity, and adaptive emotional regulation rather than merely “mental clarity.” Although these traditional concepts should not be interpreted as direct biomedical equivalents, they may enrich contemporary interpretations of stress regulation and adaptive functioning without compromising empirical rigor.

Collectively, these findings suggest that yoga-based interventions may influence resilience-related outcomes through interconnected autonomic, neuroendocrine, cognitive, emotional, and behavioral pathways, thereby providing a broader conceptual basis for their potential occupational relevance.

### 4.3 Yogic conceptualization of stress, disease, and psychophysiological regulation

Classical yogic literature conceptualizes health and disease through an integrative framework encompassing body, breath, mind, behavior, and consciousness. Within this perspective, chronic occupational stress may be interpreted not solely as a psychological disturbance but as a multidimensional dysregulation affecting psychophysiological harmony across multiple functional domains.

The yogic classification of disease described in the Yoga Vāsiṣṭha distinguishes between Ādhija Vyādhi (psychosomatically originated disorders arising primarily from mental and emotional disturbances) and Anādhija Vyādhi (disorders arising predominantly from external or non-psychological causes). In technology-driven occupational environments, persistent cognitive overload, emotional strain, sleep disruption, and techno-stress may conceptually correspond to processes underlying Ādhija Vyādhi, wherein prolonged mental disequilibrium contributes to downstream physiological dysregulation [37].

This framework may be further interpreted through the model of Pañcakośa Viveka described in the Taittirīya Upaniṣad, which conceptualizes human functioning across five interconnected dimensions: annamaya kośa (physical body), prāṇamaya kośa (vital-energy regulation), manomaya kośa (mental-emotional processes), vijñānamaya kośa (cognitive-intellectual regulation), and ānandamaya kośa (deep psychological integration and well-being). Chronic occupational stress may particularly disturb the manomaya and prāṇamaya kośas, manifesting as emotional instability, autonomic imbalance, disturbed breathing patterns, sleep dysregulation, and reduced cognitive resilience. From an integrative psychophysiological perspective, yoga-based practices may support re-regulation across these interconnected domains through breath modulation, attentional stabilization, interoceptive awareness, and autonomic balancing processes [38].

The observed effects of yoga on autonomic regulation and neuroendocrine balance may also be interpreted through traditional concepts of vāta regulation. Classical Ayurvedic and yogic frameworks describe five principal subdivisions of vāta—prāṇa, udāna, vyāna, samāna, and apāna each governing distinct physiological and psychophysiological functions. Prāṇa vāta is primarily associated with respiration, sensory integration, attention, and higher neural regulation; udāna vāta with speech, motivation, and cognitive expression; vyāna vāta with circulation and systemic autonomic coordination; samāna vāta with metabolic integration and internal homeostasis; and apāna vāta with elimination, grounding, and restorative regulation. Although these constructs should not be interpreted as direct biomedical analogues, they provide a multidimensional interpretive framework for understanding the broader psychophysiological effects of yoga-based interventions. Taken together, these conceptual models may enrich contemporary understandings of stress physiology, autonomic regulation, and resilience-related functioning while preserving the philosophical integrity of traditional yogic frameworks [39].

### 4.4 Practical and occupational implications

The present findings support the potential integration of structured yoga-based interventions within occupational health initiatives targeting technology-driven work environments. Evidence suggests that even brief and scalable interventions may support stress management, sleep quality, musculoskeletal comfort, attentional recovery, and emotional balance among employees exposed to sustained cognitive demands and prolonged screen engagement.

Within occupational settings, yoga-based “micro-practices” refer to brief structured interventions typically lasting between 2 and 10 min that can be incorporated into routine work schedules without substantially disrupting productivity [31,40]. These practices are particularly relevant for technology professionals exposed to prolonged sedentary behavior, continuous task switching, digital eye strain, and sustained attentional load. Common examples include slow diaphragmatic breathing, nāḍī śodhana (alternate nostril breathing), bhrāmarī prāṇāyāma, brief mindful-awareness exercises, seated spinal and neck mobilization practices, ocular relaxation techniques, and short guided relaxation or body-awareness pauses. Representative examples of yoga-based micro-practices for technology professionals are summarized in Table 3.

**Table 3:** Examples of yoga-based micro-practices for technology professionals.

Practice	Approximate Duration	Intended Function
Slow diaphragmatic breathing	2–5 min	Parasympathetic activation, stress reduction
Nāḍī śodhana prāṇāyāma	3–5 min	Autonomic balance, attentional regulation
Bhrāmarī prāṇāyāma	2–5 min	Reduction of mental agitation and sympathetic arousal
Seated spinal and neck stretches	3–7 min	Relief of musculoskeletal tension
Ocular relaxation/palming	2–3 min	Reduction of digital eye strain
Brief mindfulness pause	2–5 min	Cognitive reset and emotional regulation
Guided mini-relaxation	5–10 min	Reduction of fatigue and cognitive overload

Unlike conventional exercise programs requiring dedicated infrastructure and extended time commitment, workplace micro-practices are designed as brief recovery-oriented interventions embedded within routine occupational breaks or transitions between cognitively demanding tasks. Emerging evidence suggests that such interventions may support parasympathetic activation, attentional restoration, emotional regulation, and recovery from stress-related cognitive fatigue. Their brevity and adaptability may additionally improve feasibility, scalability, and adherence within hybrid and technology-driven work environments.

The increasing availability of digital health platforms also enables delivery of yoga-based interventions through online modules, mobile applications, guided audio sessions, and brief instructional videos, thereby improving accessibility for employees with demanding schedules. An occupationally adapted YT framework may therefore operate across multiple implementation levels, including: a) micro-practices lasting 2–5 min performed multiple times daily, b) structured weekly group sessions of 45–60 min incorporating gentle āsana, prāṇāyāma, and guided relaxation, and c) brief “reset protocols” of 3–7 min for acute stress regulation during high-demand work tasks.

Integration of these interventions into routine workflows, supported through digital reminders or organizational wellness initiatives, may improve adherence while minimizing disruption to productivity. Collaboration with certified yoga therapists may further enhance contextual relevance, safety, and intervention fidelity within occupational settings.

### 4.5 Evaluation strategies and methodological considerations

Future workplace interventions should incorporate validated psychological and physiological outcome measures to improve methodological rigor and comparability across studies. Recommended measures include the Perceived Stress Scale (PSS-10), Maslach Burnout Inventory, heart rate variability indices such as rMSSD and SDNN, and neuroendocrine biomarkers including cortisol. Integration of wearable technologies may further facilitate objective physiological monitoring and improve implementation fidelity in occupational settings.

Despite encouraging findings, the current evidence base remains constrained by methodological heterogeneity, relatively small sample sizes, short intervention durations, and limited long-term follow-up. Neurological correlates of resilience-related outcomes also remain insufficiently explored, and occupation-specific evidence focusing directly on technology professionals continues to be limited.

### 4.6 Critical appraisal of the evidence base

The overall quality of the included evidence should be interpreted cautiously. Although several randomized controlled trials and workplace intervention studies reported favorable psychophysiological and occupational outcomes, many investigations were constrained by relatively small sample sizes, short intervention durations, and limited follow-up periods. A substantial proportion of studies also relied predominantly on self-reported psychological measures, increasing the possibility of reporting and expectancy bias. Methodological heterogeneity across intervention protocols, occupational populations, outcome measures, and study designs further limited direct comparability across studies and precluded quantitative pooling of findings. In addition, publication bias cannot be excluded, as studies reporting positive workplace or stress-related outcomes may be more likely to reach publication. Mechanistic physiological investigations frequently involved healthy volunteers or non-occupational populations, thereby limiting direct generalizability to technology professionals. Consequently, the present findings should be interpreted as suggestive and integrative rather than definitive evidence of causal effectiveness.

### 4.7 Research gaps and future directions

Several important gaps remain within the current literature. First, most studies involve heterogeneous occupational populations, limiting specificity and generalizability to technology professionals exposed to distinct cognitive and techno-stress demands. Second, mechanistic research remains fragmented and frequently focuses on isolated biomarkers rather than integrated autonomic, neuroendocrine, inflammatory, cognitive, and behavioral pathways.

Third, longitudinal evidence remains limited, with most interventions lasting between 2 and 8 weeks and relatively few studies evaluating sustained outcomes beyond the immediate post-intervention period. Given that resilience is a dynamic and adaptive construct, longer-term follow-up extending beyond six months is warranted [41].

Implementation and translational research also remain underdeveloped. Few investigations have examined adherence patterns, organizational facilitators and barriers, leadership engagement, or feasibility within hybrid and remote work environments. In addition, classical yogic constructs such as sattva cultivation and the manas–śarīra interface remain largely absent from contemporary occupational resilience models despite their potential relevance for theoretically grounded intervention design.

### 4.8 Policy integration and organizational relevance

Integration of YT within workplace wellness programs aligns with preventive occupational mental-health strategies advocated by the World Health Organization Mental Health at Work Guidelines. Organizational support through scheduled recovery breaks, ergonomic workplace arrangements, and access to guided yoga-based sessions may facilitate normalization of stress-management practices within professional environments. Leadership engagement, institutional support, and peer participation may further improve adherence and reduce stigma surrounding workplace mental-health interventions. Within technology-driven ecosystems characterized by chronic cognitive load and sustained digital engagement, such integrative occupational-health strategies may support both employee well-being and sustainable organizational functioning.

### 4.9 Limitations

Several limitations should be considered when interpreting the findings of this review. First, the included literature was methodologically heterogeneous, encompassing randomized controlled trials, workplace interventions, mechanistic physiological studies, observational investigations, and secondary evidence sources including systematic reviews and meta-analyses. This diversity limited direct comparability across studies and precluded formal quantitative meta-analysis.

Second, many included studies involved relatively small sample sizes, short intervention durations, and reliance on self-reported psychological outcomes, factors that may increase the risk of reporting and expectancy bias. Third, occupation-specific evidence focusing exclusively on technology professionals remains limited, with several findings extrapolated from broader occupational or stress-exposed populations. Variability in intervention protocols, outcome measures, and follow-up periods may also affect interpretive consistency across studies.

In addition, integrative interpretation of classical yogic constructs alongside modern psychophysiological mechanisms remains primarily conceptual and should not be interpreted as direct biomedical equivalence. Although PRISMA 2020 reporting principles were followed, limitations related to heterogeneous evidence types and the evolving nature of occupational yoga research may affect reproducibility and comparability across studies.

Finally, conclusions regarding resilience-related outcomes, neuroendocrine modulation, autonomic regulation, and workplace effectiveness should be interpreted as preliminary and associative rather than definitive causal findings. Consequently, the present synthesis should be regarded as exploratory and integrative rather than conclusive evidence of efficacy.

## 5 Conclusion

The present review suggests that YT may represent a promising, evidence-informed, and culturally relevant approach for supporting resilience-related outcomes in technology-driven occupational settings. Across mechanistic and workplace-oriented studies, yoga-based interventions were associated with favorable trends in stress regulation, autonomic balance, emotional well-being, cognitive functioning, and occupational recovery. These multidimensional effects appear particularly relevant within contemporary work environments characterized by sustained cognitive demands, prolonged screen engagement, digital fatigue, and psychosocial stress.

Evidence from workplace studies indicates that interventions ranging from brief yoga-based micro-practices to structured multi-week programs may support reductions in perceived stress, musculoskeletal discomfort, emotional exhaustion, and sleep disturbance while contributing to attentional recovery and general well-being. The adaptability and scalability of these practices may facilitate integration into routine workplace settings without substantially disrupting productivity, thereby supporting their potential translational value in occupational health promotion.

From a traditional yogic perspective, these observations conceptually align with the cultivation of sattva, one of the three guṇas associated with psychological balance, harmony, lucidity, and adaptive self-regulation, as well as with the harmonization of prāṇa vāta involved in psychophysiological stability and mind–body integration. Within this broader interpretive framework, yoga-based interventions may support śarīra–manas–ātma saṃyoga, referring to the integrated functioning of body, mind, and consciousness that underlies adaptive resilience and well-being. Although these conceptual correspondences should not be interpreted as direct biomedical equivalence, they provide a valuable biopsychospiritual framework for understanding the multidimensional nature of resilience-related processes.

At the same time, the review highlights important opportunities for advancing the scientific and translational foundations of YT in occupational settings. Future research should prioritize occupation-specific randomized controlled trials involving technology professionals, multimodal mechanistic investigations integrating autonomic, neuroendocrine, inflammatory, and cognitive biomarkers, and longitudinal designs capable of assessing the durability of resilience-related outcomes. Greater attention should also be directed toward implementation research evaluating feasibility, digital delivery models, organizational readiness, adherence, and workforce-specific moderators within hybrid and technology-driven work environments.

Despite encouraging findings, the overall evidence base remains constrained by methodological heterogeneity, relatively small sample sizes, short intervention durations, and limited occupation-specific evidence. Furthermore, several findings were extrapolated from broader occupational and stress-exposed populations rather than exclusively from technology-sector employees. Consequently, conclusions regarding resilience enhancement, neuroendocrine modulation, and workplace effectiveness should be interpreted cautiously and viewed as preliminary rather than definitive causal evidence.

Overall, the available literature suggests that yoga-based interventions may offer a feasible and integrative strategy for supporting psychophysiological balance, adaptive functioning, and occupational well-being within increasingly demanding digital work environments.

## Funding sources

The authors declare that no funds, grants, or other support were received during the preparation of this manuscript.

## Author contributions

SKS: Conceptualization, Methodology, Software, Validation, Investigation, Writing-Original Draft, and Writing – review and editing. AKP: Methodology, Software, Formal analysis, Resources, Data Curation, and Writing-Review and Editing. PG: Validation, Writing-Review and Editing, Visualization and Supervision. All authors listed approved publication of the content and agreed to be accountable for all aspects of the work.

## Declaration of generative AI in scientific writing

During the preparation of this work the authors used Paperpal in order to grammar correction, and language refining. After using this tool, the authors reviewed and edited the content as needed and takes full responsibility for the content of the published article. The generative AI was not used to generate original scientific content, analyze data, or interpret results.

## **Declaration of conflicts ofinterest**

The authors declare that they have no known competing financial interests or personal relationships that could have appeared to influence the work reported in this paper.
