# Supplementary material for: Yoga therapy for mental resilience in technology-driven occupational settings: A qualitative systematic review and thematic synthesis
Source: J Ayurveda Integr Med. 2026 Sep 16;17(5):101394. doi: 10.1016/j.jaim.2026.101394 (PMC13599577; doi:10.1016/j.jaim.2026.101394)
Supplement: Supplementary file 1 [file mmc1.docx]

| **Database** | **Search Date** | **Search Strategy / Boolean Syntax** | **Filters Applied** |
| --- | --- | --- | --- |
| PubMed | August 2025 | (“yoga therapy” OR yoga OR pranayama OR meditation OR “mind-body intervention”) AND (“mental resilience” OR resilience OR “occupational stress” OR burnout OR “workplace stress” OR “psychological stress”) AND (“technology professionals” OR “IT employees” OR “office workers” OR “desk workers” OR “knowledge workers” OR workplace OR occupational) | English language; Human studies; Publication years 2010–2025 |
| Scopus | August 2025 | TITLE-ABS-KEY (“yoga therapy” OR yoga OR pranayama OR meditation OR “mind-body intervention”) AND TITLE-ABS-KEY (“mental resilience” OR resilience OR “occupational stress” OR burnout OR “workplace stress” OR stress regulation) AND TITLE-ABS-KEY (“technology professionals” OR “IT employees” OR “office workers” OR workplace OR occupational OR “cognitively demanding occupations”) | English language; Peer-reviewed articles; Publication years 2010–2025 |
| Google Scholar | August 2025 | (“yoga therapy” OR yoga OR pranayama) AND (“occupational stress” OR burnout OR resilience OR “mental well-being”) AND (“technology professionals” OR “IT employees” OR workplace OR occupational stress) | English language; Publications between 2010–2025; relevance-based screening |
| Manual Reference Screening | August 2025 | Reference lists of relevant systematic reviews, workplace yoga studies, and mechanistic investigations were manually screened to identify additional eligible studies not retrieved through database searches. | Peer-reviewed publications only |

Table S1. Full database search strategies used for literature retrieval
